# Naturalistic Intensities of Light at Night: A Review of the Potent Effects of Very Dim Light on Circadian Responses and Considerations for Translational Research

**DOI:** 10.3389/fneur.2021.625334

**Published:** 2021-02-01

**Authors:** Thijs J. Walbeek, Elizabeth M. Harrison, Michael R. Gorman, Gena L. Glickman

**Affiliations:** ^1^Center for Circadian Biology, University of California, San Diego, La Jolla, CA, United States; ^2^Oregon Institute of Occupational Health Sciences, Oregon Health and Science University, Portland, OR, United States; ^3^Department of Psychology, University of California, San Diego, San Diego, CA, United States; ^4^Departments of Psychiatry and Neuroscience, Uniformed Services University of the Health Sciences, Bethesda, MD, United States

**Keywords:** entrainment, physiology, translation, parametric effects, circadian, dim light, nLAN

## Abstract

In this review, we discuss the remarkable potency and potential applications of a form of light that is often overlooked in a circadian context: naturalistic levels of dim light at night (nLAN), equivalent to intensities produced by the moon and stars. It is often assumed that such low levels of light do not produce circadian responses typically associated with brighter light levels. A solid understanding of the impacts of very low light levels is complicated further by the broad use of the somewhat ambiguous term “dim light,” which has been used to describe light levels ranging seven orders of magnitude. Here, we lay out the argument that nLAN exerts potent circadian effects on numerous mammalian species, and that given conservation of anatomy and function, the efficacy of light in this range in humans warrants further investigation. We also provide recommendations for the field of chronobiological research, including minimum requirements for the measurement and reporting of light, standardization of terminology (specifically as it pertains to “dim” light), and ideas for reconsidering old data and designing new studies.

## Introduction

Light has a profound influence on the biological health of mammals, including humans. In addition to illuminating the environment for vision (i.e., image forming), light also regulates non-image forming processes, such as the circadian timing system, neuroendocrine fluctuations, and acute alerting effects, just to name a few (1, 2). All of the non-image forming effects of light studied to date appear to be most sensitive to short-wavelength light and at intensities greater than what is required for visual illumination (3). As current architectural lighting strategies have been developed around the human visual system, most do not fully support biological health and well-being. Yet, the timing of photic delivery is also of utmost importance: one major non-image forming function of light is to synchronize internal biological rhythms—orchestrated by a master pacemaker in the suprachiasmatic nucleus (SCN) in the hypothalamus—to environmental light-dark cycles. This process is called entrainment. To provide a daily photic temporal cue in lieu of exposure to the solar light dark cycle, a biologically potent light stimulus should be used to signal daytime (4), whereas a relatively less potent stimulus (dimmer and depleted in shorter wavelengths) should be utilized during the evening and night. It has been proposed that the current suboptimal patterns of photic exposure, including insufficient light during the day and excessive light at night, are at least partly responsible for a variety of negative health and safety consequences (5, 6).

Interestingly, much of the seminal work characterizing non-image forming responses to light has taken place during the biological night, yet the focus of lighting countermeasures in humans has been on optimizing photic exposure during the day. By far, the majority of these studies assesses the responses elicited by exposure to a short duration pulse of light (typically <2 h) against a background of complete darkness or dim light. Using this approach, investigators have operationally defined the lower limits of light sensitivity with respect to these biological responses to acute light pulses. A growing body of literature, however, demonstrates that light far below these putative thresholds for circadian sensitivity nevertheless exerts potent biological effects in non-human mammals, particularly if the illumination is present over several hours (7, 8).

Compared to complete darkness (to the extent that this can be feasibly created in the laboratory), dim illumination in the range approximately of moonlight and starlight markedly alters the fundamental properties of circadian rhythms in model organisms. Specifically, the addition of a relatively small number of photons throughout the biological night enhances the flexibility of behavioral entrainment by traditionally bright lighting regimens. These rarely recognized, and highly potent actions of very low light doses have been particularly well-documented in nocturnal mammals. Given the broad conservation of circadian (neuro)biology (e.g., molecular feedback loops, brain structures) and function (e.g., phase-shifting, light sensitivity) across taxa and ecological niche (see below for more detail), it is critical to ask whether comparable effects could be elicited in humans. If so, dim light treatments would represent a novel and more efficient approach for correcting or preventing circadian disturbances, particularly in more extreme cases, such as is common with jet lag and shift work. This review first aims to clarify terminology on different definitions of “dim light” and summarize the effects of very dim light in model systems. We limit the scope of our review to circadian research in mammalian organisms because of the focus on potential translational value for human circadian studies. Next, we discuss why the biological potency of very low levels of dim light should be considered and examined in humans. Although some reports showing effects of lunar cycles on human sleep exist (9–12), there are few experiments examining the effects of comparable dim light levels on circadian endpoints in humans. Therefore, we identify a range of opportunities for future human work and include recommendations for the field going forward.

## Different Levels and Definitions of Dim Light and Dark

Light levels may be reported in photometric (e.g., lux), radiometric (e.g., μW/cm^2^ or photons/cm^2^/s) and the emergent melanopic EDI (also in lux). If sufficient information is provided, which must include the spectral power distribution (SPD), these measures can all be converted into each other (13, 14). Because of very different scales, however, comparing across metrics may not always be intuitive, especially with polychromatic light. Throughout this review, we prioritize presenting measures as reported in the original work, but have converted to melanopic lux when possible and when useful for comparison.

**Figure 1:**
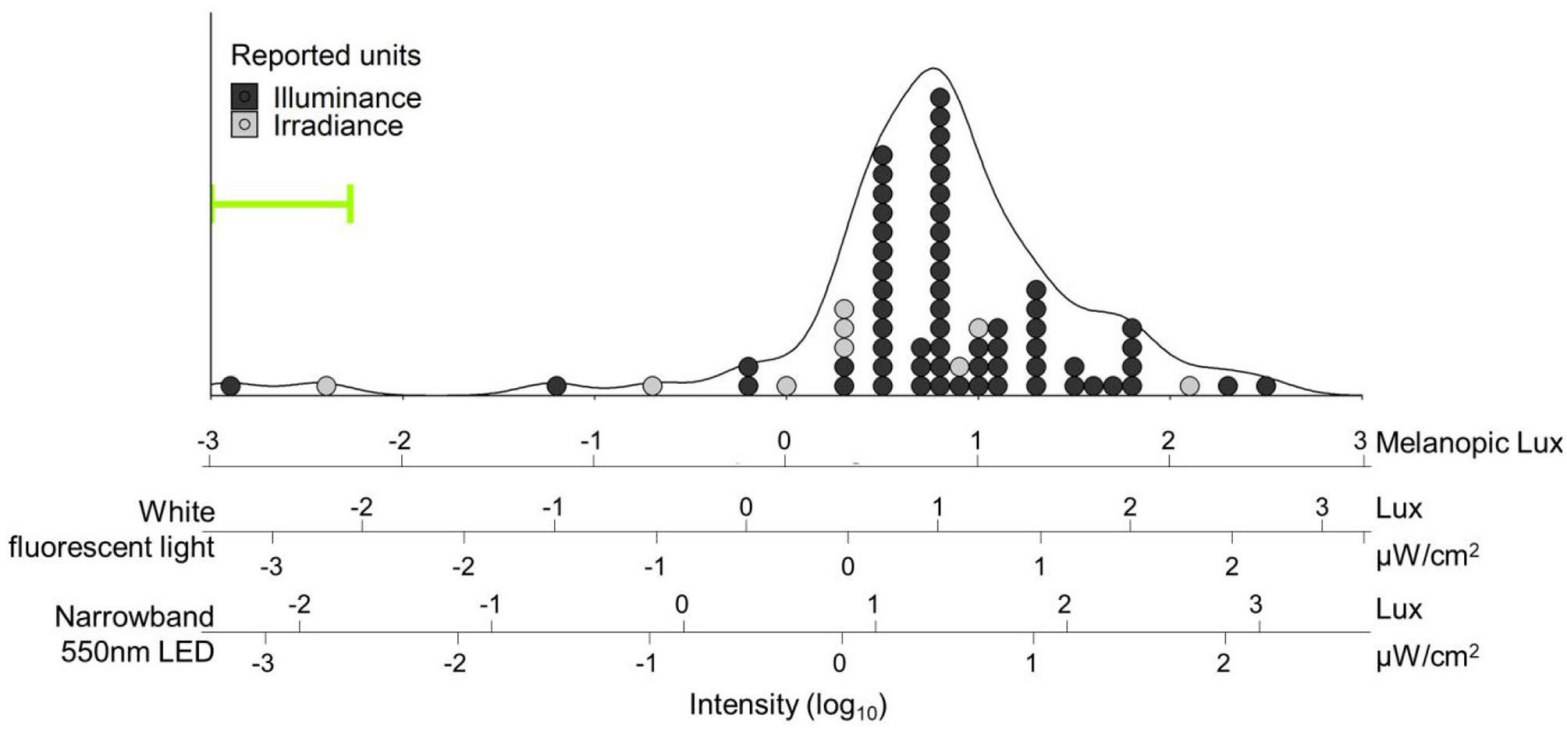
Distribution of light intensities referred to as “dim light” in human circadian research papers. Light intensities were converted into melanopic lux [using the Lucas toolbox 2014 (13)] and plotted on log units. Additional scales with white fluorescent light and narrowband green (550 ± 23 nm) light are provided and aligned for equivalent biological potency [e.g., “10 lux” (1.5 μW/cm2) narrowband green LED light has the same biological potency as 4.7 lux while fluorescent light: 2.92 melanopic lux]. Fifty-five percent of the papers (39/71) did not report any spectral or color information about their light, so SPD for fluorescent white light was assumed for conversion. Overall density is represented by the black line. Individual papers are plotted as dots, and color coded for the reported unit type; 62/71 (87%) reported light intensities in lux alone. Both mode (tallest stack) and median intensities reported as “dim light” in our search were 10 lux fluorescent white light (6.2 melanopic lux) – bright enough for phase shifting (15), entrainment (16), or melatonin suppression (17) in humans. The three papers below 0.1 melanopic lux all reported the use of red dim light; all other papers used either white light or did not report color information. Range of intensities used in Gorman lab rodent studies is indicated by the green bracket, and extends to lower values outside the bounds of the figure (see Table 1). Search strategy: A pubmed search was performed on October 23, 2020 with search terms: “human circadian dim light.” Results were filtered for “2,000-present” and “Journal Article,” which resulted in a total of 509 articles, and sorted for “Best Match.” The first 100 results were downloaded and screened for light intensity information. In total, 29 papers were excluded because they were either review papers (*n* = 11), used non-human subjects (*n* = 13), or reported neither intensity nor source (*n* = 5). From each paper the brightest light referred to as “dim light” was recorded. If the paper reported a range (e.g., 1–5 lux, or <5 lux), the highest value within the range was used. If multiple units were reported (e.g., Lux and μW/cm^2^) irradiance was used over illuminance.

Across the circadian literature, studies have used dim light for different purposes and defined a wide range of light levels as “dim” (Figure 1). These studies can generally be divided into three categories. The first group aims to eliminate any form of temporal information by exposing subjects to equal amounts of light throughout the night and day. It is worth noting that despite constant levels of environmental light, orientation of the head as well as opening and closing of the eyes inevitably leads to varied photic exposure patterns. In humans, dim constant light is more commonly used to study intrinsic clock properties that are not directly driven (or masked) by light (20).

The second body of work aims to mimic urban levels of artificial light at night to study its effects on sleep or health (21, 22). These studies usually focus on the ramifications of our modern lifestyle and the ubiquity of artificial light on natural processes, such as exposure to electronic devices, indoor LED lighting, or light derived from artificial light sources outside, such as street lights. Thus, such studies typically use 5–30 lux during the “night,” as opposed to near darkness. These protocols lead to all kinds of health decrements, including loss of sleep and metabolic syndrome in both rodents [reviewed in (23)] and humans [reviewed in (24)].

The last group of studies uses much lower levels of light (commonly 0.01–0.1 lux) that are more consistent with natural light at night (nLAN) cast by the moon or stars (9–12, 25–38). In contrast to the relatively brighter light used in constant light or studies of urban nighttime light, nLAN seemingly does not induce circadian health disruption, though data are limited. It does, however, have marked effects on circadian organization of behavior, despite being reported as below threshold for phase shifting or melatonin suppression. These changes in circadian behavior, which generally indicate a higher degree of circadian flexibility, have so far been primarily studied in rodents, but yield strong translational potential in aiding the adjustment of the human circadian clock to either rapid travel across multiple time zones or irregular schedules such as in shiftwork.

To illustrate the wide range of light levels referred to as “dim light” by circadian researchers, we performed a literature review and gathered light parameters summarized in Figure 1 (see legend for search methodology). When reviewing these articles, we noticed that, often, investigators reported extensive detail about the bright light stimuli employed in their studies but did not quantify or qualify the “dim light.” Specifically, 39/71 (55%) only reported “dim light” levels in lux (only relevant for the image-forming system), without any information on spectral quality or source; a further 5/76 (6%) were excluded from our analyses because they did not even report intensity.

Because absolute darkness in the laboratory often cannot be feasibly achieved or measured with any confidence, it is difficult to define precisely where the “threshold” is between (very) dim light and darkness. Walls, doors and ceilings are rarely completely light-tight, and equipment in or near animal housing may also generate light. The QA-4 modules in the VitalView data collection system commonly used for circadian monitoring, for example, have red indicator lights that fluctuate when an activity signal is received and can produce feedback (39). Moreover, investigators may intentionally put in place constant, low levels of illumination for practical reasons, such as animal husbandry (6), sometimes without reporting. Careful exclusion or attenuation of all such light sources can, of course, diminish light exposure.

It is our impression that many circadian researchers share the assumption that (very) dim light is biologically equivalent to actual darkness. Such beliefs might be founded on carefully executed experiments demonstrating minimal light levels that are required to induce phase shifts or melatonin suppression (40–42). As we will lay out in this review, however, there is a multitude of evidence, within the circadian literature, as well as from other fields, that light well below 1 lux can have dramatic effects on circadian oscillators or their outputs, challenging these common assumptions. By summarizing these (perhaps unintentionally) overlooked effects of very dim light levels within circadian research, we hope to provide a framework to aid researchers in designing and reporting on their experimental lighting conditions.

## Historical View of nLAN Studies

Laboratory studies cannot escape trade-offs between simplicity and experimental control on one hand and real-word validity on the other. In the domain of circadian entrainment, this trade-off has been operationally resolved in favor of exposure to an alternation between just two photic conditions, each of unvarying spectrum, intensity and geometry, to represent “day” and “night.” Twilight transitions, lunar cycles, and variability due to cloud cover are just a few examples that reflect naturally-occurring changes in intensity and spectrum that are rarely simulated, although each of these photic dimensions may be highly relevant to circadian timing systems of particular organisms, and it could be adaptive for such organisms to be sensitive to them (43). The work in the 1960's of zoologist JL Kavanau (44–46) on rest/activity cycles of mammals provided myriad demonstrations of the behavioral importance of additional domains of photic stimuli. For example, running speed, perhaps a reflection of motivation, was sensitive to light intensity during twilight; rodents of various genera operantly manipulated light intensity in relation to their endogenous rest/activity cycles; and animals expressed reliable (but non-homogenous) orientation preferences toward light sources, such as always running toward a dim night-time light source, conceptualized by the investigator as a simulated moon. In short, there emerged a rich ecology of environmental lighting that did not, for whatever historical reason, strongly imprint on the emergent field of chronobiology, though the zoological tradition remains important (30, 37, 47).

## Serendipitous Discovery of nLAN Effects in an Experimental Setting

It came as a great surprise when we accidentally “rediscovered” the fundamental importance of dim illumination on circadian entrainment, when we thought we knew it to be of little consequence. In preparing for a time series collection of Syrian hamster (*Mesocricetus auratus*) brains for a study of SCN rhythmicity in a paradigm termed “bifurcation,” we discovered notable and nearly categorical entrainment differences between the first and second cohort of animals. We had thought that each set of 20 hamsters was exposed to identical conditions, which included a very dim green light that was continuously illuminated—day and night—to aid in working with animals at night. These lights (see Table 1 for intensities) had been incorporated after thorough (but perhaps not thorough enough) consultation of the literature suggested their inefficacy (e.g., thresholds for melatonin suppression and phase shifting) and some further experimental validations that they did not induce classic circadian responses. Confronted by the statistically improbable result that chance alone caused animals 1–20 to respond one way and animals 21–40 another, we looked for other explanations, only to discover that the electrical power for dim nocturnal illumination had been disrupted in half of the animals. Although we approached this explanation with skepticism, a *de novo*-test confirmed a potent entrainment role of this very dim light source (48). Since that time, we have come to appreciate that the effects of nLAN are more generalizable than we initially imagined.

**Table 1:** Range of reported Gorman lab nLAN values.

**560 + 23 nm LED**	**Lowest reported value (18)**	**Highest reported intensity (19)**
Illuminance (Lux)^a^^,^^b^	0.004	0.03
Irradiance (μW/cm^2^)	0.00062	0.00390
Photon Flux (Photons/cm^2^/s)	1.75 × 10^9^	1.09 × 10^10^
S Cone (α opic lux)^a^^,^^b^	0.0000001	0.000014
M Cone (α opic lux)^a^^,^^b^	0.00367	0.02370
L Cone (α opic lux)^a^^,^^b^	0.00352	0.02150
Rod (α opic lux)^a^	0.00193	0.01440
Melanopsin (α opic lux)^a^	0.000614	0.0053

a *Values are calculated based on reported irradiance using the Lucas toolbox 2014 (13)*.

b *Values based on human cone sensitivity*.

Demonstrations of a potent circadian role for nLAN both preceded and ran parallel to our work [reviewed in (30, 37, 47)]. Working with bats, for example, Erkert demonstrated systematic variation in circadian entrainment parameters with variations in light intensity in the range of starlight (32–35, 49). Readers interested in the ecological breadth of the biological effects of nighttime light could probably not do better than to consult a comprehensive resource, such as that from Rich and Longcore (47), or this recent review (50). But in the circadian domain specifically, our lab has accrued a critical mass of studies contrasting fundamental properties of circadian rhythms in animals under dimly illuminated vs. conditions without detectable light. We present a synthesis here that is not easily derived from reading the publications as they individually appeared.

### Range of Parameters Used in our Lab Over the Years

Over the years, we have used a variety of nLAN sources. Our initial studies assessed the efficacy of a single green narrowband LED (560 nm, full width at half maximum of 23 nm) mounted on the wall of a light-tight enclosure containing a single opaque plastic cage that provided some diffusion. Other studies employed panelescent LED nightlights ~1 m from free-standing caging racks, with animals housed in clear cages. Most of our studies have used a single green LED mounted in a standard position ~10–30 cm from the orthogonally oriented running wheel in transparent cages (Table 1).

Unlike highly controlled human studies that can employ Ganzfeld domes, pharmacological pupil dilation etc., the animal housing environment allows ample opportunity for variation in effective light exposure. Although in various enclosures we have taken pains to minimize variability in light exposure via diffusers, positioning of cages, etc., conventionally, we have reported light intensity at the brightest location in the cage. But as intensity falls off by the inverse square law, it can vary considerably within the cage. Moreover, animals oriented directly toward the light [as in Kavanau's rodents (44)] might, depending on the experimental setup, be sampling several orders of magnitude more light than animals with their gaze diverted from the point source. Conversely, effective illumination of the retina could be attenuated with pupillary constriction.

In short, it is not yet possible to define with any precision the range of parameters of nLAN that produce a substantial effect on rodent rest/activity cycles, but the sources and configurations used do not appear to be near the limits of an operating range. Effects appear to be robust with respect to some variation in caging configuration and light orientation, although systematic assessments of these factors have not been completed. Finally, the narrowband green light is likely to activate multiple photoreceptor mechanisms. In limited studies, white light proved also to have efficacy, so we have little reason to believe that narrowband stimulation is a requirement, although this remains to be thoroughly assessed.

Despite a variety of enclosures as well as light sources and intensities (see Table 1) used throughout two decades of experiments to investigate the effect of simulated natural night light levels, the nLAN conditions were always deliberately contrasted with darkness, created by careful exclusion or attenuation of all light sources. As mentioned above, typical laboratory conditions do not achieve complete darkness. In our lab, we made every effort to exclude light to approximate complete darkness. Even after covering all equipment LEDs with black electrical tape and blocking all visible light coming into the room through doors or ventilation, however, our dark conditions may not be absolute. Nevertheless, they were always below the limits of detection (using an IL1700 radiometer; International Light, Inc., Newburyport, MA).

## How Does nLAN Affect Circadian Rhythms in Model Systems?

Historically, the actions of light on circadian systems have been commonly categorized as either parametric or non-parametric. A non-parametric effect is exemplified by an acute resetting of circadian phase induced by a brief light pulse, and an absence of any sustained changes in any other parameters of the underlying clock oscillation. Such effects are the basis for much entrainment theory, particularly as it applies to nocturnal animals that are exposed to short intervals of bright light at dawn and dusk. Parametric actions, in contrast, are typified by ongoing modulations of clock function, such as a change in the free-running period, often caused by chronic light exposure. In reality, this conceptual distinction can be problematic, because parametric and non-parametric effects cannot always be completely distinguished. A brief light pulse, for example, can both acutely reset circadian phase and alter free-running period (51). Further, shifts in the light:dark cycle induce rapid shifts in the phase of activity but the SCN oscillatory network may be perturbed for several cycles before returning to steady state (52, 53).

Circadian rhythms in behavior are described using several common metrics, including period and amplitude. Even though different circadian assays are often used to measure one or more of these metrics, basic circadian theory suggests that these parameters are interrelated. Here we describe the effects of nLAN on some of the most common circadian measures. Throughout this review, we will use “nLAN” to refer to light levels below one lux, whether it is used to illuminate strictly during the night, as a constant condition, or as a brief pulse, and regardless of the source. For an overview of definitions of the various parameters, see these reviews: (54–56).

### Effects of nLAN on Common Rhythm Parameters

#### Period

Period is the time it takes for the circadian oscillator to complete one cycle. The period of the internal circadian pacemaker can only be measured in conditions absent external time cues (zeitgebers) such as light or temperature cycles. Across multiple species, nLAN changes the free-running period (8, 25, 57, 58). For example, Syrian hamsters exposed to continuous complete darkness have a free running period (FRP) of just under 24 h. In contrast, in constant dim illumination (<0.01 lux, nLAN) these same animals have FRPs of ~0.3 h longer (8). These findings are consistent with one of “Aschoff's rules” (more accurately characterized as generalizations), which notes the tendency of FRPs to increase with light intensity in nocturnal rodents (59, 60).

#### Waveform

Each full cycle in behavioral rhythms can be divided into biological day and night, which can be assessed by monitoring the relative lengths of an organism's active (alpha, α) and rest (rho, ρ) phases. In nocturnal rodents, alpha and rho correspond to (biological) night and (biological) day, respectively, and vice versa in diurnal humans. Although less commonly assayed and reported than is free-running period, waveform (relative length of alpha and rho) is a fundamental property of a circadian rhythm and is encoded in the SCN (56, 61–65). Aschoff observed that alpha in nocturnal rodents tends to decrease with increasing light intensity. In stark contradiction of this rule, the active phase of Syrian hamsters increases by 3 h in nLAN vs. darkness (8). Effects of nLAN on alpha are discernible even under entrained conditions with varying photoperiods. In most nocturnal species examined, exposure to long winter nights – or short days – eventually yields a waveform with an elongated biological night as assessed by lengthened activity duration, melatonin secretion or SCN pattern of electrical firing and gene expression (61, 66). Upon transfer of male Siberian (*Phodopus sungorus*) and Syrian hamsters from summer to winter photoperiods, nLAN accelerates short-photoperiod entrainment as well as its downstream sequelae (e.g., photoperiodic regulation of reproduction) (8, 67–70).

#### Amplitude

Whereas the free-running rhythm of a measured rhythm unambiguously reflects the period of an underlying pacemaker, the same is not necessarily true for rhythm amplitude. The amplitude of a rhythm describes the magnitude of the change in an output signal (for example body temperature, wheel-running or SCN electrical firing) throughout one cycle. Mathematically, pacemaker amplitude has been conceptualized as related to its phase perturbability by external zeitgebers, with small amplitude pacemakers having larger amplitude phase shifting responses (71, 72). Paradoxically, short photoperiods increase the amplitude of phase shifting by bright light pulses in rodents (68, 69), suggesting a smaller pacemaker amplitude while simultaneously increasing the daily variation in SCN electrical firing (73, 74). With respect to nLAN, there is sporadic evidence of increased wheel-running amplitude in Syrian hamsters (48). In subsequent studies using slightly different running wheels, the amount of wheel-running was indistinguishable in dark vs. nLAN conditions (8). To our knowledge, wheel-running amplitude has never been observed to decrease with nLAN. Importantly from the perspective that wheel-running intensity may have feedback effects on circadian pacemakers (75), the matched activity levels in dark vs. dim nLAN excludes this feedback as the basis for observed differences in circadian period and waveform among male Syrian hamsters (8). In other contexts, nLAN-effects can be documented in the complete absence of running wheels (70).

Together, characterization of basic circadian parameters clearly demonstrates that feasibly attainable levels of darkness (e.g., <10^−6^ lux) and nLAN are not biologically equivalent despite the common belief that such “dim” intensities fall below the threshold for markedly altering circadian function.

### nLAN as a Zeitgeber

#### Dim Light PRC

Brief exposure to light (i.e., light pulses) can induce phase shifts in free running organisms. Depending on the timing of these light pulses, the phase shifts can be either advancing (shifting earlier), delaying (shifting later), or of minimal magnitude. The relationship between timing of the stimulus and the resulting phase shifts is described in a phase-response curve (PRC). While the amplitude of the light PRC can vary depending on the intensity, duration, and spectral properties of light, the shape is very consistent across species (76). Typically, light induces advances at the end of the (biological) night, delays at the beginning of the biological night, and minimal phase shifts during the middle of the biological day, in both diurnal and nocturnal animals. Against a dark background, 2-h pulses of nLAN in male Syrian hamsters yielded a statistically significant PRC with an amplitude of 45 min that was generally similar in shape to that from a 15-min bright light PRC (8).

#### Entrainment

The ability of nLAN light pulses to induce phase shifts suggests that such illumination might be a strong enough zeitgeber to entrain mammals. The Gorman lab at UC San Diego, however, has never directly tested entrainment in a light-dark cycle alternating nLAN and complete darkness (dim-dark). Efforts by others, on the other hand, did demonstrate that mice (C57Bl/6) exposed to 12 h dim−12 h dark light cycles were able to entrain with lights as dim as 0.0005 lux, depending on the wavelength (77). Similarly, Molossid bats (*Molossus molossus*) were able to entrain to 0.1 lux, with half of the animals entraining to light as dim as 0.00001 lux (33). Lastly, daily 2 h pulses of 0.07 lux of “deep red” light was enough to reliably entrain female albino rats (78). In sum, even relatively short exposure to nLAN levels of light can be a zeitgeber in at least four different species.

### Acute Effects of nLAN

In addition to affecting the underlying circadian pacemaker as described above, low levels of dim light can affect the output behaviors directly.

#### Masking + Melatonin Suppression

Masking refers to the phenomena where a stimulus directly suppresses (negative masking) or enhances (positive masking) a certain behavior below or above levels expected based on circadian phase. These changes are direct effects on clock outputs and might not necessarily change the underlying circadian pacemaker. In rodents, a common example of negative masking is the suppression of activity levels by light during the biological night. Positive masking is often seen following a cage change during the day, where activity levels are temporarily higher than typical for the time of day because of novelty-induced activity that does not reflect the underlying circadian biology (79). Common examples of masking in humans are activity-induced changes in core body temperature, light-induced increased alertness, or suppression of plasma melatonin levels by light. Even if masked responses do not necessarily originate from or affect the underlying circadian pacemaker (although in the example of light-suppressed activity in rodents or melatonin in humans, it might do both), masking is important to every circadian researcher as it might affect inferences made about the underlying pacemaker if not appropriately controlled (80, 81). Furthermore, masked behavioral or physiological signals may feed back to the core clock and alter it in subsequent cycles.

In rodents, thresholds are different for positive (increased activity by darkness) and negative (decreased activity by light) masking, and depend on wavelength (79, 82–86). In mice, the lowest intensity at which positive and negative masking was observed with 518 nm light was 10^9^ and 10^12^ photon flux, respectively (77). In Siberian hamsters, nLAN was sufficient to induce strong positive and negative masking, depending on the timing and duration, although effects cannot be completely separated from entrainment effects (87).

Melatonin suppression, often considered a form of negative masking, is the most commonly measured effect of light in human research (88) and relies on the SCN (66). The threshold intensity to acutely suppress pineal melatonin in Syrian hamsters was determined to be between 0.019 and 0.186 μW/cm^2^, or 0.11 and 1.08 lux (89), similar to a lower threshold for 300 s 503 nm light of 3.5 × 10^10^ photons/cm^2^/s (41). In an entrainment study in Syrian hamsters, 8 h of nLAN (0.1 lux) significantly suppressed melatonin expression compared to darkness (8). Because 2 or 5 h of nLAN was not sufficient to suppress melatonin in the same animals, the effects seen with 8 h of exposure might result from differences in entrainment itself, rather than acute suppression (masking). Similarly, in young Sprague-Dawley rats maintained in light-dark cycles with various amounts of dim light at night, 0.08 μW/cm^2^ (0.2 lux) light suppressed plasma melatonin, while 0.06 μW/cm^2^ did not (90), and in female nude rats, 0.08 μW/cm^2^ (0.2 lux) of light at night was sufficient to attenuate nightly melatonin levels (91). Again, entrainment effects cannot be separated from acute masking. Although the relative contribution of acute and chronic exposure to (or parametric vs. non-parametric effects of) nLAN might not be definitive, it should be apparent that nLAN affects both levels of behavior and melatonin in a variety of rodent species.

#### Pupil Responses

Because many circadian responses are slow, difficult, invasive, and/or expensive to measure, pupillometry is commonly used as a proxy for circadian response. Light-induced pupil constriction relies on the same non-image forming visual system (e.g., photoreceptors and synaptic connections) as the circadian clock, and can therefore be a useful substitute psychophysical measure (92). Just like most measures, pupillary light responses are graded responses that depend on intensity and wavelength. In mice, reliable pupil constrictions were observed at 10^10.4^, 10^11.4^, and 10^12.4^ photon flux for 470, 517, and 626 nm light, respectively (77). These thresholds are roughly comparable to levels reported by others (93–95), corroborating the overall conclusion that nLAN elicits biologically relevant responses (77, 96).

### nLAN as a Potentiator for Other Stimuli

The research summarized above demonstrates that very low levels of light influence both internal circadian rhythms as well as the behavioral and physiological outputs we measure to infer internal circadian conditions. Furthermore, nLAN can interact with, or potentiate, other light and non-visual stimuli. In this section, the research on indirect effects of nLAN is summarized.

#### Phase Angle of Entrainment

A phase angle is the relative timing between two phase markers. For the phase angle of entrainment in nocturnal animals, the timing of onset of activity is often expressed relative to lights-off transition. Non-parametric entrainment predicts that oscillators with different free-running rhythms entrain to the same zeitgeber signal with different phase angles of entrainment (97). Following the lengthening of FRP with dim light, this would predict a relatively later activity onset in a light-dim compared to a light-dark cycle. On the contrary, activity onset was observed to occur 0.8 h earlier in Siberian hamsters exposed to nLAN compared to dark controls (87).

#### Resetting

The phase response curve of a 15-min bright light (100 lux) pulse in male Syrian hamsters free-running in low levels of dim light does not statistically differ in shape or amplitude from the same PRC obtained in constant darkness (8). Notwithstanding this similar bright-light PRC, nLAN does facilitate re-entrainment following a phase shift (simulated jet-lag experiment) (87, 98). For example, in both Syrian and Siberian hamsters, nLAN significantly accelerated re-entrainment recovery following a 4- or 8-h phase shift compared to completely dark nights, and was observed for advances as well as delays and among animals of different ages. In the most robust case, animals exposed to dim light re-aligned activity up to 68% faster (98).

#### Resetting to Non-photic Cues

While light is the most potent and dominant zeitgeber for mammalian circadian rhythms, non-photic cues can also phase shift and/or entrain rodent behavior. For example, introducing a wheel-naive animal to a running wheel triggers activity. The phase shift that can be induced by this novel wheel running is amplified by nLAN (99). Similarly, a cage change can induce activity levels atypical for the circadian phase which produces a significant PRC. Amplitude of the PRC of cage-changing, however, was not significantly altered by nLAN (8).

#### Extreme Entrainment: T-Cycles and Bifurcation

Non-parametric entrainment theory explains entrainment by positing daily discrete phase corrections of the same magnitude as the difference between the internal and external rhythms. Real-life entrainment, however, is undoubtedly more complex than just a daily non-parametric phase resetting (100, 101). As mentioned above, the shape of the bright light PRC was not affected by an nLAN background, but re-entrainment to simulated jetlag is reliably accelerated. This observation indicates that nLAN influences re-etrainment by bright light regimes in a manner that goes beyond simple non-parametric resetting.

A near-defining feature of circadian rhythms is their narrow range of entrainment. In most mammals, entrainment to light-dark cycles much more than 2 h longer or shorter than 24 h would be considered exceptional. This range, however, can be very markedly expanded by incorporation of nLAN and/or twilight transitions. Once again, Kavanau raised the possibility of such an effect, but provided only sketchy data in support of the claim (46). More definitively, in a study of twilight transition effects in Syrian hamsters, Boulus et al. (102) demonstrated a markedly increased range of entrainment to both short and long (non-24 h) cycles (T-cycles), compared to typical instantaneous transitions between day and night conditions; however, besides ramping up or down the intensity gradually, the experimental manipulations also included a tonic exposure to nLAN throughout most of the scotophase. Without explicitly engaging the issue of this nighttime illumination, the authors attributed the enhanced range of entrainment to the twilight transitions. Having demonstrated tremendous efficacy of nLAN, we assessed whether its presence, without twilight transitions, was sufficient to extend the upper range of entrainment (we did not conduct a parallel assessment of short T-cycles). Indeed, the upper range of entrainment was significantly extended with nLAN, and some animals met entrainment criteria at cycles as long as 30 h (103). Using dim light at night, various rodent species have demonstrated reliable entrainment to cycles as extreme as 6 h on either side of 24 h (19, 103–105). This type of circadian flexibility is unprecedented in genetically intact mammals with dark nights.

As referenced above, nLAN facilitates a bifurcated pattern of entrainment in mice and two species of hamsters exposed to particular 24 h LDLD cycles, which of course is not a zeitgeber condition experienced in nature. For example, mice (C57Bl/6j) exposed to LDLD 7:5:7:5 with dark nights will generally entrain with a unimodal pattern of wheel running and treat the alternate scotophases as biological day (remain mainly inactive). In the presence of nLAN, however, most mice reorganize their behavior and divide activity between the two scotophases nearly symmetrically. Although some T24 LDLD conditions could be equivalently described as a T12 LD cycle, non-T12 LDLD conditions such as LDLD9:5:5:5 etc. also induce and maintain bifurcation. Systematic investigations of bifurcated entrainment clearly establish that it reflects temporally reorganized 24 h underlying oscillations rather than a 12 h clock (48, 106–110).

Both of these consequences of nLAN – enhanced T-cycle entrainment and behavioral rhythm bifurcation – cannot be explained by simple masking (19, 104, 106, 110–119). By the same token, neither entrainment pattern is readily understood by non-parametric entrainment theory that accurately models entrainment to more traditional experimental regimens. For example, the extended upper range of entrainment in nLAN is not explicable in terms of a proportionately increased delay portion of the light pulse PRC, nor a sufficiently large lengthening of free-running period (beyond the 0.3 h effect described above). Phase angles in dim-facilitated entrainment in non-24 h lighting conditions do not consistently follow the patterns predicted by relative length of the LD-cycle and the FRP, suggesting more complex entrainment mechanisms must play a role (19, 104, 112, 120). In addition, the acceleration of re-entrainment following simulated jetlag, which occurs for phase advances and delays of various sizes, cannot be explained by changes in free-running period without concomitant changes in the PRC amplitude.

The mechanisms by which nLAN affects circadian behavior remain to be much more deeply investigated, but nLAN is known to acutely affect electrical activity in the SCN. Specifically, electrophysiological recordings in awake Wistar rats show acute responses in SCN cellular activity to light levels as low as 0.15 lux (lowest intensity reported) (121). Additionally, some nLAN effects are mediated by the intergeniculate leaflet (122), a thalamic area with neuropeptide Y projections to the SCN and which has been implicated in integration of non-visual feedback to the SCN (75). Despite the lack of a clear mechanistic understanding, a recurring theme, beyond the scope of this review, is that nLAN may alter coupling interactions among multiple oscillators comprising the circadian pacemaker (67, 123, 124), and this change in coupling results in a more flexibly entrained oscillator to a host of conditions. Coupling remains a poorly understood dimension of circadian organization, but it is central to the functioning of a complex pacemaker. It is worth mentioning that the behavioral effects of nLAN appear often to be categorical (e.g., bifurcated or not) rather than modest extensions of entrainment parameters based on bright light entrainment theory. Thus, nLAN seems to be doing something fundamentally different than tweaking known dose-response relationships between light and phase-shifting. Collectively, these experiments demonstrate that in the right conditions, the circadian system can be much more flexible than traditional circadian theory predicts. Such flexibility is unprecedented in intact animals without genetic or pharmacological intervention or with completely dark nights.

## Comparing Rodents to Humans

To properly assess how relevant the summarized research on nLAN in model organisms is for human circadian rhythms, in this section we compare the pertinent neurobiology across species. First, the mammalian circadian system is very well-conserved (76, 125), including fundamental properties of the primary oscillators. While the main body of mechanistic basic research involved nocturnal rodents (mice, rats, hamsters), circadian rhythms have been well-described in diurnal rodent species, including grass rats, degus and squirrels as well as larger mammals, including sheep and several species of non-human primates [e.g., baboon (126)]. In humans, the possibilities for mechanistic, anatomical, molecular, or neuronal studies are limited, but available data from post-mortem studies, for example, [e.g., (127–129)], confirm homology with other species. Circadian rhythms in all these species rely on a similar transcription-translation feedback loop and a functionally equivalent retinohypothalamic tract, and are orchestrated by an anatomically similar SCN with comparable cell subtypes (127, 130). Furthermore, human circadian rhythms in behavior and physiology match those of other mammalian species rather closely. Functionally, rhythms in the SCN and melatonin levels (as well as suppression and resetting by light) are similarly phased in nocturnal and diurnal species, including humans, (i.e., they are tied to environmental day/night rather than the rest/activity cycle) (131–133). Because of the similarities in circadian clock functions across species (76, 134, 135), the presumption should be that the human biological clock might, as in rodents, exhibit un(der)studied responses to low levels of light that are generally accepted to be ineffective. We will discuss the field's use of dim and dark as a control in more depth below.

Biological potency of light does not solely depend on the molecular and neurological foundations in the SCN, but also on the retina. Most mammals have three classes of photoreceptors (136). Cones are the photoreceptors used for color vision under photopic conditions (brighter light) and are the most variable between species. While primates, including humans, have three types of cones (S, M, and L, for short, medium and long wavelengths, respectively) (137), mice only have two types, lacking a long-wavelength cone found in primates and rendering mice incapable of distinguishing between green and red light (138). Albeit with less sensitivity, mice can still respond to long-wavelength light and are not blind to “red light” [reviewed in (96)]. In primates, 99% of cones are in the fovea, providing us with high-acuity vision in a small part of our visual field (139). Mice also lack a fovea, making the entire retina much more similar to the peripheral retina in primates. The majority of photoreceptors, in both primates and rodents, are the much more sensitive rods, which are primarily at play under low light conditions (140, 141). Because a primate eye is bigger than a mouse eye, the total number of photoreceptors is larger in primates. Both cones and rods have been demonstrated to contribute to circadian responses to light (142–144).

Furthermore, the mammalian retina contains a third type of photoreceptor: intrinsically photoreceptive retinal ganglion cells, or ipRGCs (143, 145–148). The much more recently-discovered ipRGCs are present in both humans and rodents, are highly conserved across species (149, 150) and are primarily responsible for the majority of non-image forming photic responses (85). In addition to their intrinsic photoreception through melanopsin, ipRGCs also receive input from other photoreceptor types (151). Combined intrinsic and extrinsic input makes ipRGCs sensitive, albeit with an increased latency, to nLAN lower than 10^7^ photons/cm^2^/s, close to absolute detection limits in human vision (146, 152). This suggests that nLAN effects rely at least in part on rod-mediated photoreception, which may be routed through ipRGCs. The similarities in these two types of photoreceptors between mammalian species [review: (153, 154)] corroborate the idea that humans could be sensitive to such light levels as well.

Even if retinal circuitry for dim light reception were comparable between rodents and primates, one might question whether the inverted rest/activity cycles would render dim light at night irrelevant to diurnal animals who are sleeping with closed eyelids through much of the night. First, there is no data in rodents suggesting dim light needs to be delivered uninterrupted throughout the night. In fact, outside, under natural conditions, rodents might spend parts of their night underground, only experiencing ecological light at night during a limited window (30, 155, 156). Second, light has been delivered in sleeping humans (157–159), demonstrating that closed eyes do not fully block light or its effects on circadian biology. For example, although the purpose of the experiment was to study traditional bright light responses, brief pulses of light flashed through closed eyes while sleeping induced significant phase shifts in melatonin rhythms (159). Total transmission through adult eyelids is estimated at 0.3–3%, with more transmission for longer wavelengths (160, 161). Even if this means intensities for sleeping humans may need to be adjusted to correct for closed eyes, given the range of light levels described in nLAN studies (0.01–0.1 lux), that would still include very dim light (<3 lux), significantly below intensities of most night lights and electronic devices as well as most “dim” light used in human studies (Figure 1).

Together, comparisons of circadian neurobiology and ocular neuroanatomy provide ample evidence to believe the effects of nLAN in rodents could be relevant to humans, and that they would not be impractical or unfeasible as targets of circadian manipulation. While closed eyelids are a valid factor to consider when optimizing delivery of light, we believe the cross-species similarities are strong enough to warrant careful study of the effects of nLAN in humans.

## Dim Light Studies in Humans

Most physiological effects of light, albeit not all (e.g., alpha, as discussed above), in both humans and model systems, demonstrate a characteristic intensity-dependent response, with increasing photic intensities yielding relatively greater responses. Examining the magnitude of these responses across a range of light intensities allows for the construction of a fluence response curve, with a reportable goodness of fit of the data to a sigmoid function, which provides various parameters of interest. Threshold sensitivity represents the lowest intensity of light required to elicit a detectable physiological response, and saturation occurs at the intensity at which additional light cannot increase the response further. The photic dose eliciting a half-maximum response (ED_50_) is also a useful point of comparison, as it occurs along the steep portion of the curve, comfortably within the range of responsiveness. Consequently, when relatively lower light levels are capable of eliciting an equivalent (ED_50_) response, greater photic sensitivity can be inferred.

In comparing human fluence-response curves for phase shifting and melatonin suppression to those in some of the most commonly employed model systems [but see (162) for greater taxonomic consideration], there appear to be species differences in photic sensitivity, as indicated by significantly lower values of ED_50_ in hamsters 0.04–1.64 melanopic lux (41, 68, 163) vs. humans ~4–60 melanopic lux (40, 42, 164–168) (see Table 2). When human studies did not optimize other elements known to exert an influence, such as spectrum and pupil dilation, relatively greater intensities of light (60–75 melanopic lux) were required to achieve a comparable half-saturation response, and this is true across different physiological effects of light (2, 40). Yet, the light levels required to elicit a response in those studies are also more likely to map on to real world applications, where white polychromatic light exposures to eyes with freely responding pupils are the norm. Most animal work with complete fluence-response curves has not optimized spectrum or pupil dilation and thus, the ~2 orders of magnitude difference in ED_50_s between species is likely to represent an underestimation. Nonetheless, the 560 nm narrowband nLAN stimulus typically used to illuminate the scotophase in Gorman lab experiments is orders of magnitude lower than the calculated ED_50_ (or even minimum threshold) for melatonin suppression in the same species. By the same logic, even after considering species differences in ED_50_, humans might be sensitive to light well below 1 lux. Therefore, the presented effects of nLAN underscore the idea that light in the tail of the fluence response curves could still be potent enough to elicit circadian responses, but might be overlooked by focusing on effects at ED_50_.

**Table 2:** ED_50_ for melatonin suppression and phase shifting in humans and hamsters.

	**Wavelength (nm)**	**FWHM^a^ (nm)**	**Photon flux (Photons/cm^**2**^/sec)**	**Photopic lux**	**Melanopic lux**
**Melatonin suppression**					
Syrian hamster^b^ (41)	503	20	10^10.11^	0.01	0.04
Human^c^ (42)	555	10–14	10^12.94^	21.14	4.39
**Phase shifting**					
Syrian hamster^b^ (41)	503	20	10^11.49^	0.33	0.93
Human^c^ (42)	555	10–14	10^13.07^	28.57	5.85

a *Full width at half maximum*.

b *Study employed a 5 min light pulse*.

c *Study employed a 6.5 h light pulse*.

Most commonly, dim light in the nLAN (or even brighter) range of intensities appears in human studies only as a control condition and/or in contrast to a “bright light” condition [e.g., (15, 169)—see Figure 1]. But the assumption that there is no effect of nLAN on circadian physiology should be recognized as such—an assumption. First, without a no-light control group, we cannot say so definitively. Second, given the fact that ipRGCs also contribute to brightness discrimination (149), it is quite conceivable that a circadian response to a light stimulus might be affected by the contrast (fold increase) with the background, or light history, rather than absolute intensity. For example, switching between a background of 0.1 and 1 lux could conceivably be as important as the difference between a 100 and 1,000 lux stimulus. Lastly, there is emerging evidence that the precise mechanisms and pathways differ across various circadian responses (69, 77, 170–174). This also applies to the shape of the fluence response curve—for example, while effects of light may be largely linear for phase-shifting or melatonin suppression, the effects of nLAN, which appear to be largely parametric, are likely non-linear. The field of circadian research is littered with designs based on these assumptions, whether implicit or explicit.

In classic studies of the physiological effects of light in humans, participants are often maintained under dim light conditions or darkness prior to administration of a light pulse in order to control for the potential effects of photic history. Though the intensity and duration of that period of adaptation varies across studies, research that has systematically examined the effects of photic history in humans has shown robust effects. Most of these studies have altered daytime photic exposure and then, examined subsequent response to a test pulse during the biological night. For example, Chang et al. (169, 175) had participants spend 3 days in the laboratory under either dim light (1 lux) or relatively brighter light (90 lux) during waking hours, while scotopic levels were kept very dim (<0.1 lux) across both conditions. When participants were subsequently exposed to a 6.5 h light pulse (90 lux) ~1 h prior to habitual bedtime, a 40 min greater circadian phase delay and increased alertness occurred with pre-exposure to daytime dim vs. brighter light. Earlier studies similarly had participants exposed to more or less daytime light in the week preceding a bright light test pulse during the biological night, and there was significantly increased melatonin suppression and phase shifting after the dimmer daytime condition (176–178). In contrast, within a single, much shorter time frame (2 h), early in the scotophase and during the biological night, dim light served to attenuate the melatonin suppression response to a subsequent 90 min test pulse as compared to a completely dark adaptation period (179). Together, these findings are consistent with the recommendation of a high day-night contrast, in terms of biological potency, in order to clearly signal time of day to the circadian timing system and maximize physiological responses to light [e.g., (14, 180)]; however, they leave open the question as to whether complete darkness or nLAN is an optimal scotophase condition.

Two unique examples of studies that included human exposure to nLAN include work from Wright et al. (181, 182), in which participants were exposed to only natural sources of light (including moon and starlight) while tent camping. In the first paper, circadian outputs for this week were compared, within participants, to a more typical week that included work, school, self-selected sleep schedules, and time spent in built environments with electrical lighting. The smaller phase angle of entrainment to the solar day during the camping period of only natural light exposure was primarily attributed to less electrical light at night and more light (from the sun) during the day, yet nLAN cannot be discounted as a potential contributing factor. Indeed, in the second study, nocturnal light while camping is characterized in more detail, and they report sensor-derived night light levels in the 500–600 nm range between 0.1 and 1 lux (in addition to intermittent light from a campfire) (182).

To our knowledge, there has never been a direct examination of the effects of very dim vs. complete darkness throughout the scotophase in human studies of the physiological effects of light. Considering the varied background conditions in the seminal human work on the topic, it may be possible to retrospectively compare a variety of “dark” scotophase conditions across experiments in order to glean whether or not there are potential effects of nLAN in humans that are similar to what has been established in model systems.

## Summary and Recommendations to the Field

While it remains to be rigorously examined, there is good reason to suspect that the potent effects of very dim light (akin to moon and starlight intensities) summarized here have translational potential for human circadian research, including the fact that the tremendous overlap in circadian physiology and responses between species make it likely that such effects are possible in humans. However, even if these effects do not directly translate (i.e., if it is established later that these lower levels of light do not markedly affect the human circadian system in these ways, or indeed, in any discernible way), they nevertheless provide a window into the potential for a latent plasticity that may exist in humans and may be inducible via other mechanisms, which have yet to be determined (119). Keeping both of these notions in mind, but focusing on the former, the following section includes some recommendations for future work for circadian researchers focused on mammalian systems.

### Measurement and Reporting of Light

This review focuses on photic intensity; however, light can vary along several other important dimensions, including spectral properties, duration, and directionality. All of these parameters have been shown to affect biological potency. Recent progress has been made, via an internationally balloted consensus-based process, in developing a standardized method of measuring and reporting light for non-image forming physiological effects of light [CIE S 026:2018 (14)]. Essentially, the intensity for each of the five **α** -opic photoreceptors (S-cone-opic, M-cone-opic, L-cone-opic, Rhodopic; Melanopic) is determined, allowing for the assessment of the relative contribution to a given response; this is conceptually similar to and builds upon the introduction of melanopic lux (13). Under typical lighting conditions, melanopic contribution is the dominant photoreceptor mediating photic input for the physiological effects of light in humans, such as melatonin suppression and phase shifting (183–185). Thus, when quantifying photic stimuli for these physiological effects of light, stimuli should be characterized in terms of melanopic Equivalent Daylight Illuminance (EDI), which is also reported in units of lux (14). While we support these new metrics for quantification of light for non-visual responses, converting previously reported photopic lux values into precise melanopic lux/EDI is often not feasible due to insufficient information about the spectral quality of the light stimulus. The Lucas and CIE toolboxes (13, 14), however, support such conversions by providing standardized or estimated SPD for several light sources. (Figure 1; see below for recommendations for reporting light in research papers). Furthermore, the primary purpose of this review is to emphasize the potency of nLAN, which has largely been assumed to be subthreshold for affecting the mammalian circadian system, regardless of spectrum. The research summarized above should make it abundantly clear that light levels multiple orders of magnitude below 1 lux are sufficient to alter circadian rhythms both directly and indirectly.

In all work going forward, researchers should take pains to ensure that lighting conditions are reproducible and convertible. This requires detailed reporting of the set up, the distance and gaze of the organism, placement of sensors, the measurement instruments used (including their ranges of accuracy, the exact sensor-heads used, the ability to set a 0-reference point, etc.), and any other factor that could influence the effect of light (e.g., directionality, dilated pupils, etc.), see (186). Many labs, however, are likely ill-equipped to accurately measure light levels in the nLAN range. At a minimum, researchers should state that light levels were below levels of detection by their equipment, state what those levels are, and report details on any effort taken to reduce light levels, such as removing or blocking light sources. The availability of open access and supplemental options mean a much more detailed account of lighting set ups need not be subject to space or word limit concerns. At a minimum, the specifications of the light source need to be described. For example, light sources that can be described as “white light” or “white fluorescent” can span at least an order of magnitude in their biological potency (183, 187). Further, as discussed above, light levels should be reported in units that can be later converted (e.g., spectral power distributions of all light sources) if reporting metrics change, as is likely as more is learned (14). For example, the bulk of the nLAN work described here was conducted before the emergence of melanopic lux (13, 14). However, because the spectral composition and irradiance are reported in our work, we can now calculate the melanopic lux, or any other metric that emerges from the field subsequently (see Table 1 and Figure 1).

### Standardization of Terminology

Standardization of language both within the circadian and lighting communities surrounding light levels, especially lower levels, is crucial, both in our science communications as well as public health outreach and messaging. In the future, it might be useful to develop a set of agreed upon terms to further narrow the range of intensities currently termed as “dim light.” For example, “dim” has been used variously to describe values between 0.0001 and 500 lux (see Figure 1), which spans many orders of magnitude. In this paper, we have used the term “nLAN” throughout to describe intensities of light no brighter than naturally occurring nighttime light levels.

As a field, our current recommendation that “nights should be completely dark,” may warrant further consideration. Importantly, solar night is not naturally completely dark, and while the total absence of light is perhaps a more simple aim than prescribing a sweet-spot range of dim photic intensities that do more good than harm, absolutely no light at night may not always be feasible, optimal, or desirable. Furthermore, the lighting industry may play an important role in helping to optimize not just our days but our nights as well, with the development of novel technologies that capitalize on these more nuanced understandings.

### Reconsidering Old Data and Designing New Studies

Existing data sets and conclusions drawn from them may be reconsidered in light of the evidence presented here. It may be possible to conduct a systematic review and/or meta-analysis of data from near-scotopic conditions, either within or across species.

Additionally, while painstaking, new studies of the biological effects of light in humans need to include sufficient data points to measure responses at different intensities, including no detectable light, and time courses.

Recent discoveries show that not all physiological effects of light rely on identical mechanisms [e.g., alertness vs. melatonin suppression (41, 42, 69, 77, 85, 95, 170–174)]. Thus, ED_50_s of one circadian response should not be assumed to generalize to a different response. The same caution applies to sensitivity values reflected in the tails of fluence response curves, where estimation is less reliable. To better understand these response differences, further studies aiming to better understand relative photoreceptor contribution and to identify practically important dose response parameters should ideally include measurements across multiple responses (e.g., pupillary constriction, entrainment) in the same subject under the same experimental conditions. Where collection of multiple endpoints might not be feasible, the use of standardized, and replicable, methodologies becomes even more critical to enable between study comparisons.

Finally, should investigators need dim illumination for practical purposes, given the relative biological potency of short wavelengths, including at relatively “dim” levels (Figure 1), we recommend the use of the dimmest light, most-depleted of short wavelengths, possible. For example, 10 lux with a 650 nm LED, more than is needed for comfortable vision, is only 0.01 melanopic lux, as opposed to 10 lux fluorescent light (~3,000 K), which is 6.2 melanopic lux (Figure 1). While the former light source is undoubtedly less potent, researchers should nevertheless bear in mind that, in at least one case, “deep red” light levels as low as 0.07 lux were sufficient to elicit behavioral entrainment in albino rats (78). Furthermore, this same lighting stimulus was described by the authors as insufficient to aid researchers with animal handling. Thus, as with many experimental design considerations, there are trade-offs between rigor and practicality; however, there may be no such thing as “dim enough” when trying to minimize effects of background light – any amount of light may potentially affect the circadian system. Therefore, investigators should both consider and report light levels in every condition, including those that may have otherwise been simply described as “dim” or “dark.”

## Author Contributions

GG, TW, EH, and MG: conceptualization. TW, EH, and GG: analysis. TW, EH, GG, and MG: writing. GG: funding acquisition. All authors contributed to the article and approved the submitted version.

## Conflict of Interest

The authors declare that the research was conducted in the absence of any commercial or financial relationships that could be construed as a potential conflict of interest.
